# TFE3-expressing primary perivascular epithelioid cell tumor of the Lymph node mimicking nodal relapse of rectal cancer: A case report

**DOI:** 10.1016/j.ijscr.2019.05.002

**Published:** 2019-05-11

**Authors:** Jongmin Park, An Na Seo

**Affiliations:** a Department of Radiology, Central Physical Examination Office, Daegu, South Korea; b Department of Pathology, School of Medicine, Kyungpook National University, Daegu, South Korea; c Department of Pathology, Kyungpook National University Chilgok Hospital, Daegu, South Korea

**Keywords:** LN, lymph node, PEComa, perivascular epithelioid cell tumor, TFE3, transcription factor E3, TSC, tuberous sclerosis complex, Lymph node, Perivascular epithelioid cell tumour, Rectal cancer, Relapse, Transcription factor E3

## Abstract

**Introduction:**

Perivascular epithelioid cell tumor (PEComa) is a rare mesenchymal neoplasm.

**Presentation of case:**

We treated a 50-year-old woman who underwent neoadjuvant chemoradiotherapy followed by low anterior resection and adjuvant chemotherapy for ystage IIIB rectal adenocarcinoma. The patient was diagnosed with metastatic rectal adenocarcinoma in the right lower lobe of the lung after 14 months of follow-up. Later, after 3 years of follow-up, a mass in the gastrosplenic area was revealed by computed tomography, which was considered indicative of metachronous mass relapse. The patient underwent partial omentectomy, and an enlarged mass measuring 2.2 × 1.3 cm was found. Microscopic findings revealed an alveolar-like collection of epithelioid cells surrounded by a thin-wall vascular channel within a lymph node (LN). These epithelioid cells were positive for HMB45 and TFE3 but negative for Melan-A, CK, and S100. The patient was finally diagnosed with TFE3-expressing primary PEComa of LN.

**Discussion:**

We report a rare case of TFE3-expressing primary LN PEComa that mimicked nodal recurrence of rectal cancer. Thus, imaging results in patients affected by neoplasm present a clinical diagnostic dilemma, as the presence of newly onset PEComa of LN may lead to the erroneous diagnosis of tumor recurrence or progression.

**Conclusion:**

Suspicious isolated recurrences of rectal cancer in unusual locations may require surgical excision to confirm definitive diagnosis.

## Highlights

• TFE3-expressing PEComa has unique morphological and immunohistochemical features.
• PEComa can mimic lymph node relapse of rectal cancer.
• Surgical excision can aid a proper diagnosis of suspicious lesions in rectal cancer.

## 1 Introduction

Perivascular epithelioid cell tumor (PEComa) is a mesenchymal neoplasm characterized by distinct histological features and immunophenotype [1]. This tumor was first recognized as a separate entity in the 2002 World Health Organization classification. PEComas are composed of epithelioid cells with clear to eosinophilic cytoplasm and spindle cells demonstrating melanocytic and smooth muscle differentiation. PEComas most frequently develop in the retroperitoneum, abdominopelvic region, uterus, and gastrointestinal tract [1,2]. Tuberous sclerosis complex (*TSC*) 1/2 gene is commonly inactivated in conventional PEComas of either syndromic or sporadic type [3,4]. Recently, a distinct subset of PEComas with transcription factor E3 (*TFE3*) rearrangement, unique morphology, and characteristic immunophenotype has been revealed [4]. It has been suggested that PEComa with *TFE3* rearrangement may present with malignant histological features and exhibit a relatively more aggressive clinical behavior than conventional PEComa.

We herein report an illustrative case of TFE3-expressing primary PEComa of a lymph node (LN) in the gastrosplenic area that mimicked nodal relapse from rectal cancer as suggested by imaging. This work has been reported in line with the SCARE guidelines [5].

## 2 Case report

**Fig. 1:**
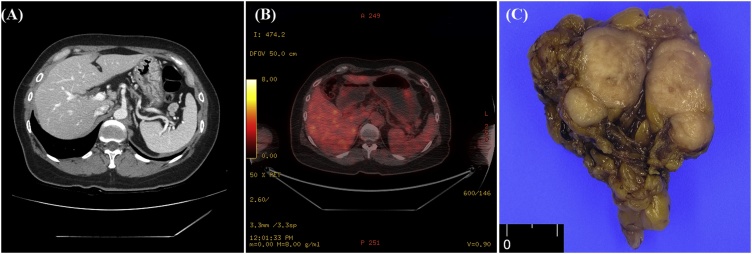
(A) Computed tomography findings of the abdominopelvis. Several enlarged lymph nodes are noted at the gastrosplenic area. (B) ^18^F-fluorodeoxyglucose positron emission tomography identified mild uptake (standardized uptake value max 2.8) in the gastrosplenic area. (C) Gross examination showed oval solid mass measuring 2.2 × 1.3 cm.

**Fig. 2:**
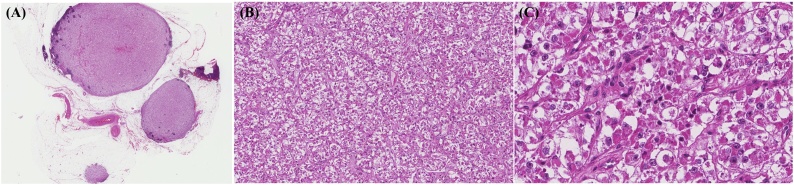
(A) Representative scan view of hematoxylin and eosin staining of the resected specimen (taken at 0.4× objective). (B) Epithelioid cells were arranged in a nested or alveolus-like architecture with surrounding branching thin-walled vascular spaces (taken at 10× objective) (C) The individual epithelioid cells show clear to eosinophilic granular cytoplasm with prominent nucleus and mitoses was also found (taken at 40× objective).

**Fig. 3:**
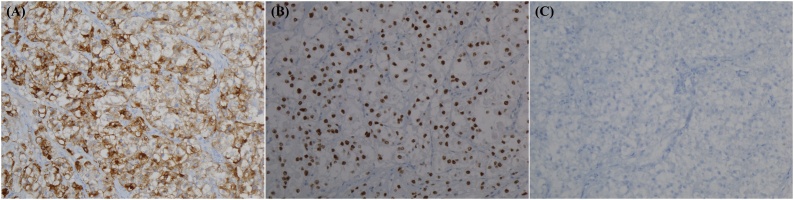
Representative imaging for immunohistochemical staining. Tumor cells showed (A) strong cytoplasmic expression for HMB45 and (B) strong nuclear expression for TFE3. (C) In contrast, tumor cells showed no expression for Melan-A (all images were taken with a 20× objective lens).

A 50-year-old woman who was diagnosed with ypT3N1bM0 (stage III) moderately differentiated adenocarcinoma of the rectum was treated with a low anterior resection after preoperative chemoradiotherapy and adjuvant chemotherapy in 2013. The follow-up included clinical examination, serum carcinoembryonic antigen measurements every 3 months and abdominopelvic and chest computed tomography scan every 6 months for the first 2 years. After 14 months of the follow-up, she developed a recurrence of rectal adenocarcinoma in the right lower lobe of the lung and underwent curative wedge resection. Additionally, the patient was treated with eight cycles of XELOX (oxaliplatin and capecitabine). At the abdominopelvic computed tomography scan made in December 2015, several enlarged LNs were found in the gastrosplenic area (Fig. 1A). ^18^F-fluorodeoxyglucose positron emission tomography identified mild uptake (standardized uptake value max 2.8) in the gastrosplenic area (Fig. 1B), which was highly suspicious of rectal cancer relapse. At that time, the level of carcinoembryonic antigen, a tumor marker, was normal (0.78 ng/mL). To confirm this diagnosis, laparoscopic partial omentectomy was performed to remove a splenic hilum nodule. Gross examination revealed a well-encapsulated and lobulated mass 2.2 × 1.3 cm in size. The cut surface showed homogeneously solid and brown color (Fig. 1C). Microscopic findings showed a thick fibrous capsule in the periphery of the nodule with some LN features (Fig. 2A). Epithelioid cells occupied most of the LN, being arranged in a nested or alveolus-like architecture supported by branching thin-walled vascular spaces and/or delicate collagenous stroma (Fig. 2B). Individual epithelioid cells had clear to granular eosinophilic cytoplasm (Fig. 2C). Tumor cell nuclei were round to ovoid shape; mild atypia and prominent nucleolus were found. Spindle cells were not observed. Multinuclear tumor cells were seen infrequently. Melanin pigment granules were infrequently noted in tumor cell cytoplasm. Prominent necrosis was not observed, and mitotic activity was counted at up to 4 of 10 high-power fields (HPFs). In immunohistochemical studies, HMB45 and TFE3 were strongly and diffusely expressed (Fig. 3A and B). In contrast, Melan-A (Fig. 3C), smooth muscle actin, CK, and S100 were not expressed. On the basis of these histological and immunohistochemical findings, the diagnosis of PEComa with TFE3 expression was considered. We classified our case according to the Folpe’s classification of PEComas [6], and this neoplasm was classified as “benign”. The patient did not receive additional adjuvant chemotherapy for this neoplasm. After 36 months following surgical excision, the patient remained healthy with no evidence of recurrence in the clinical and radiological follow-up. This study was approved by the Institutional Review Board of Kyungpook National University Chilgok Hospital (No. 2019-02-012).

## 3 Discussion

PEComas are mesenchymal tumors that consist of distinctive cells that show melanocytic and smooth-muscle differentiation. The PEComa family includes angiomyolipoma, lymphangioleiomyomatosis, clear cell “sugar” tumor of the lung, and a group of morphologically and immunophenotypically similar tumors developing in various soft tissues and visceral sites [2]. PEComas are associated with genetic alterations similar to those in TSC, an autosomal dominant genetic disease caused by the loss of *TSC1* (9q34) or *TSC2* (16p13.3) genes. Their protein products regulate the Rheb/mTOR/p70S6K pathway [7]. Recently, Argani et al. suggested that PEComas harboring *TFE3* gene fusions might be a distinctive entity [8]. In addition, an accumulating body of evidence indicated that PEComas with *TFE3*-rearrangement have distinct morphology and immunophenotype compared to those of conventional PEComa [3,8]. PEComas are composed entirely of clear epithelioid cells with nested or alveolar-architecture, round to ovoid nuclei, strong expression of TFE3 and HMB45, and minimal expression of muscle markers [3]. Furthermore, such PEComas lack *TSC1*/*TSC2* inactivating mutations of conventional PEComas and retain their protein expression according to immunohistochemistry data [7].

The *TFE3* gene is located on the short arm of the X chromosome and encodes a member of the helix-loop-helix domain-containing transcription factor family that binds MUE3-type E-box sequences in gene promoters [9]. Tumors with *TFE3* rearrangement include PEComa with *TFE3*-rearrangement, Xp11-associated renal cell carcinoma (now reclassified as MiT family translocation renal cell carcinoma), melanotic Xp11 neoplasm, and alveolar soft part sarcoma [1,3].

Unfortunately, neither normal counterparts nor precursor lesions for PEComas have been unknown. In this regard, it should be noted that by examining 80,677 LNs systematically dissected for gynecological malignancies, Nagasaka et al. showed that minute PEC nests preferentially developed in para-aortic and high pelvic LNs [10]. Based on these findings, they suggested that soft tissue PEComas in the retroperitoneum and abdominopelvic region may emerge from LNs [10]. In our case, epithelioid cells with alveolar architecture detected within the tumor showed distinct features of LN. Therefore, LN-arising PEComa represents a clinical issue in the patients affected by neoplasm that typically spreads to lymphatic channels: in such cases, the presence of a newly onset PEComa of LN may lead to the erroneous diagnosis of tumor recurrence and progression if only imaging results are taken into account.

Most PEComas are benign, even though some exhibit aggressive behavior [11]. Firm malignancy criteria in PEComa have not been established because metastasis and recurrence have been exceptionally rare for this tumor [1]. Folpe et al. proposed that PEComas should be considered malignant if they show two or more worrisome features (>5 cm, infiltrative growth pattern, high nuclear grade and cellularity, >1 mitotic counts/50 HPF, necrosis, and vascular invasion) [6]. Clinically aggressive PEComas spread to metastatic sites, including the lung, liver, LNs, and bone. Because histological characteristics of our case showed only one such feature (four mitotic counts/10 HPF), it was considered as benign. However, accumulating evidence suggests that PEComas with *TFE3* rearrangement have relatively aggressive clinical behaviors [3,12].

Generally, in conventional advanced PEComas pathologically activated by the loss of the *TSC1*/*TSC2* tumor suppressor complex, mTOR1 is a rational mechanistic target for the therapy with its inhibitors, such as rapamycin or everolimus [13]. However, patients with *TFE3*-rearranged PEComas, which do not involve the *TSC2* gene, will likely fail to respond to mTOR1 inhibitors [14]. Therefore, evaluation of *TFE3* gene expression in PEComas could recognize this entity and aid therapeutic decisions.

## 4 Conclusion

We report an intriguing case of TFE3-expressing primary PEComa of the LN mimicking nodal recurrence of rectal cancer as suggested by imaging data. Although metastasis of the primary rectal cancer is most concerning, suspicious isolated recurrences of the primary tumor in unusual locations may indicate other potential causes. Surgical excision can be a viable option for confirm diagnosis and avoid inappropriate treatment.

## Conflict of interest

No potential conflict of interest relevant to this article was reported.

## Sources of funding

None.

## Ethical approval

Ethically approved (IRB No. 2019-02-012).

## Consent

Written informed consent was obtained from the patient for publication of this case report and accompanying images.

## Author’s contribution

Substantial contributions to the conception or design of the work: An Na Seo.

The analysis, or interpretation of data for the work: Jongmin Park.

Drafting the work or manuscript writing: Jongmin Park and An Na Seo.

Final approval of the version to be published: An Na Seo.

## Registration of research studies

This is a case report not a research study and, therefore, registration of research studies is not required.

## Guarantor

Dr. An Na Seo.

## Provenance and peer review

Not commissioned, externally peer reviewed.
